# Probiotic supplements for relieving stress in healthy participants: A protocol for systematic review and meta-analysis of randomized controlled trials

**DOI:** 10.1097/MD.0000000000015416

**Published:** 2019-05-17

**Authors:** Ning Zhang, Xing Liao, Yanan Zhang, Menglin Li, Weiguang Wang, Shuangqing Zhai

**Affiliations:** a School of Traditional Chinese Medicine, Beijing University of Chinese Medicine; b Center of Evidence Based Traditional Chinese Medicine, Institute of Basic Research in Clinical Medicine, China Academy of Chinese Medical Sciences; c Jinzhou Hospital of Traditional Chinese Medicine, China.

**Keywords:** meta-analysis, probiotic supplements, protocol, psychological stress, systematic review

## Abstract

**Background:**

Psychological stress is a ubiquitous subjectively negative emotional experience, but excessive psychological stress has adverse effects on the happiness in our lives and physical and mental health, and may cause many health problems. Studies have found that probiotics have a certain role in alleviating negative emotions, reducing abnormal behaviors, improving cognitive function, and also showing the great potential of probiotics in relieving psychological stress. At present, many clinical trials have been carried out to intervene in populations with psychological stress with probiotic supplements, but there still lack of targeted systematic review and meta-analysis.

**Methods and analysis:**

The Cochrane Central Register of Controlled Trials, Embase, MEDLINE, Psycoinfo, Cumulative Index to Nursing and Allied Health Literature will be searched to obtain the eligible randomized controlled trials published up to March 1, 2019. Meanwhile, the references to relevant publications will also be reviewed to identify other studies, and will re-search before finial summary for analysis. EndNote X7 will be used as a document manager for duplicate checking and screening of literature. The risk of bias will be assessed and the date of included studies will be analyzed by Revman V5.3.5.

**Results:**

The primary outcome will be subjective stress level, general mild psychiatric symptoms of participants. The secondary outcome will be cortisol level and adverse effects likely to be related to treatment.

**Conclusion:**

The systematic review and meta-analysis will provide evidence to assess the efficacy and safety of probiotics in relieving psychological stress.

**PROSPERO registration number:**

PROSPERO CRD42019122930.

## 1 Introduction

### 1.1 Description of the condition

When an individual perceives that demands from society, work and study tax, or exceed his or her adaptive capacity, psychological stress unavoidably occurs.^[1]^ For the human being, psychological stress is more than a subjectively negative experience.^[2]^ Generally, stress response is important for enhancing adaptability and coping with threatening situations.^[3]^ However, excessive psychological stress is negative,^[4]^ not only increases the risk of diseases, included hypertension,^[5]^ cardiovascular diseases,^[6]^ digestive system diseases,^[7]^ and most neuropsychiatric disorders^[8,9]^, but also may cause reduced happiness in life,^[10]^ job burnout,^[11]^ unhealthy lifestyles,^[12,13]^ and other consequences. Statistics showed that in the UK, at least one-third of work-related diseases are caused by stress, resulting in a large loss of working time.^[14]^ Therefore, finding an effective means to prevent stress, relieve stress, and reduce the adverse effects of stress on individuals, families, and society has become a hot issue in the present research, especially in the medical field.

### 1.2 Description of intervention

A large number of diverse intestinal microbes are planted in the human gut, which are symbiotic with the host and participates extensively in the life activities of the host.^[15,16]^ Studies have found that^[17–19]^ intestinal microbes can regulate the mood, cognition, and nervous system function of the host through various means such as nerve, immunity, and endocrine (ie, brain-gut axis). Stress is an important factor in the intestinal micro-ecological disorders, and may cause more health problems.^[17]^

Probiotics are active micro-organisms. When applied in sufficient quantities, probiotics can play a beneficial role by improving the intestinal tract for ecological balance.^[20]^ Animal and human studies have confirmed that in some cases, probiotics can increase or decrease the synthesis of certain neurotransmitters and biologically active factors such as serotonin,^[21]^ brain-derived neurotrophic factor,^[22]^ cortisol,^[23]^ thereby alleviating the subjective stress level of the participants, as well as related mental symptoms such as anxiety and depression. Because probiotics have a positive role in mood, cognition, and other psychological processes, probiotics are also known as “psychobiotics”^[24,25]^ and may be a potential therapy or auxiliary means for stress-related mental disorders.

### 1.3 Why this systematic review is needed

So far, a large number of clinical studies on the application of probiotic supplements to intervene in stress response have been carried out worldwide. However, the Cochrane Database of Systematic Reviews, Embase, and MEDLINE databases were searched, and evidenced reviews or protocols on “whether probiotics have effects on relieving psychological stress in healthy participants” were not found. Therefore, this systematic review is urgently needed.

## 2 Methods

### 2.1 Study registration

The protocol has been registered in PROSPERO, the International Prospective Register of Systematic Reviews with registration number CRD42019122930 on February 21, 2019.

### 2.2 Ethics and dissemination

Ethical approval is not necessary as this paper is a reanalysis of the original study, which does not involve participations’ privacy issues. The results of this study will provide information on the effectiveness and safety of probiotics supplements to alleviate psychological stress in healthy populations. This protocol will be disseminated by a peer-reviewed journal, thus providing evidences for clinical practice.

### 2.3 Participants

The healthy participants with no known major health issues, regardless of age, gender, ethnicity, and region will be included. Pregnant women will be excluded in the selection criteria for this study due to potential adverse effects.

### 2.4 Interventions and comparisons

Studies that use oral probiotic supplements as interventions will be included. The probiotics can be in the form of tablets, powders, capsules, soft capsules, fermented milk, or fortified foods containing probiotics. Studies in which probiotics do not survive, (eg, after heat-killed) or use prebiotics as intervention alone will be excluded.

Trials will be included if they use placebo as a control. If compared with the control group, the effect of probiotics alone can be used in the treatment group, for example using probiotic yogurt and normal yogurt as comparison, will also be eligible.

### 2.5 Types of studies

All randomized controlled trials that reporting subjective stress level of participants will be included. If mean deviation and standard deviation of the study results cannot be obtained, then the study will be excluded. If the results of a study are reported multiple times, we will combine these reports and consider them as 1 study.

### 2.6 Outcome measures

#### 2.6.1 Primary outcomes

(1) Subjective stress level: measured using the Perceived Stress Scale, Berocca Stress Index, Personal Strain Questionnaire of the Occupational Stress Inventory-Revised, or Visual Analog Scales, and so on.
(2) General mild psychiatric symptoms: measured using the General Health Questionnaire, Psychological General Well-Being Schedule, State/Energy Visual Analogue Scales, Hospital Anxiety and Depression Scale, Hamilton Anxiety Rating Scale, Depression Anxiety Stress Scale, Visual Analog Scales, Geriatric Depression Scale, or Hopkins Symptom Checklist-90, and so on.

#### 2.6.2 Secondary outcomes

(1) Cortisol level (saliva, plasma or serum, and so on);
(2) Adverse effects likely to be related to treatment.

### 2.7 Search strategy

Cochrane Central Register of Controlled Trials, MEDLINE, EMBASE, PsycINFO, and Cumulative Index to Nursing and Allied Health Literature will be searched, and the references to relevant publications will also be retrieved to identify additional studies. The searches will be re-run before the final analyses. The detailed search strategy is seen in Tables 1 to 5.

**Table 1:**
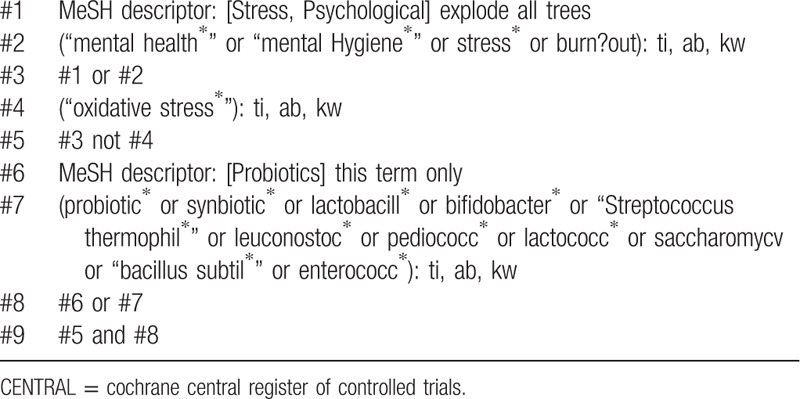
CENTRAL search strategy.

**Table 2:**
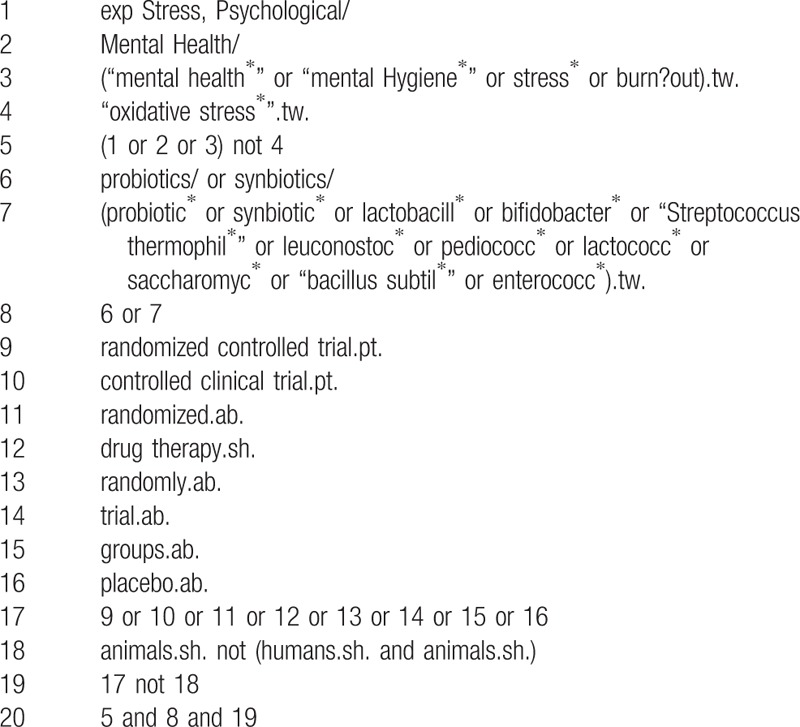
Medline search strategy through Ovid.

**Table 3:**
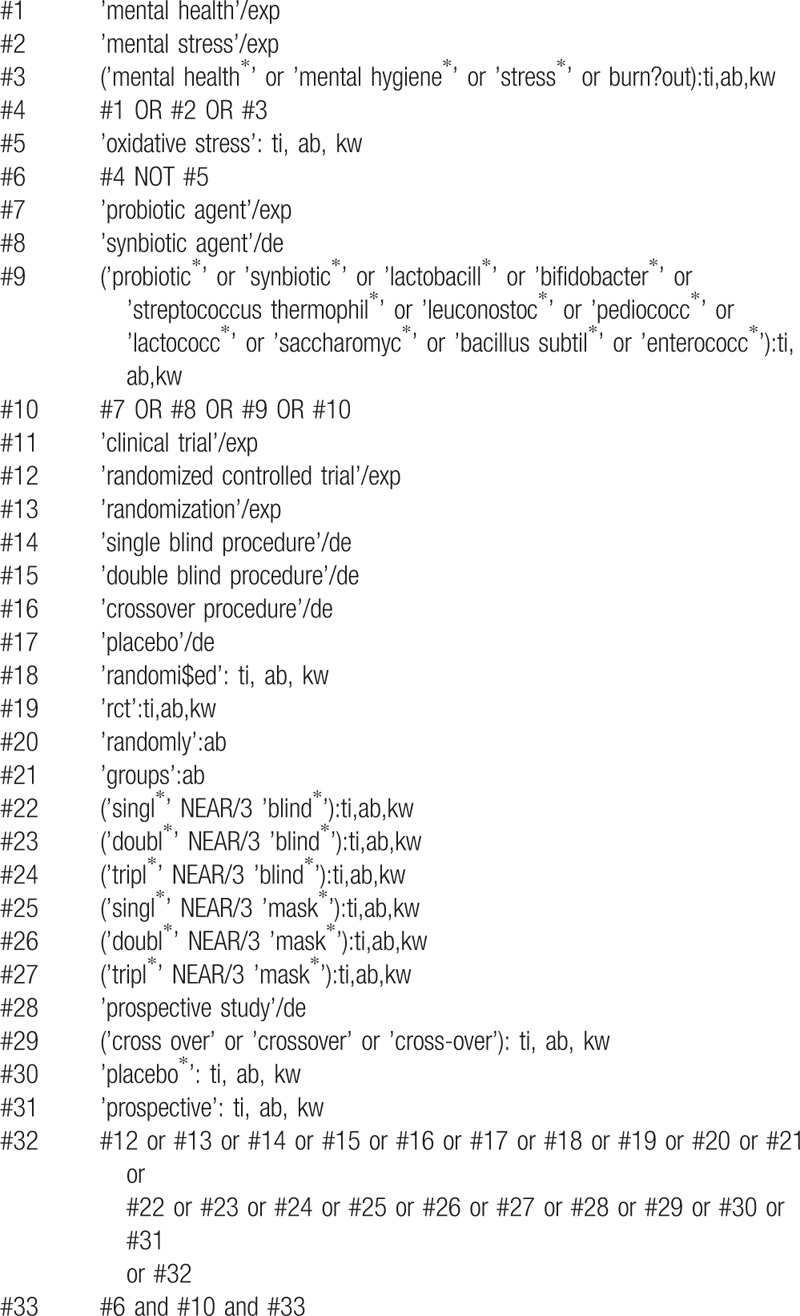
Embase search strategy.

**Table 4:**
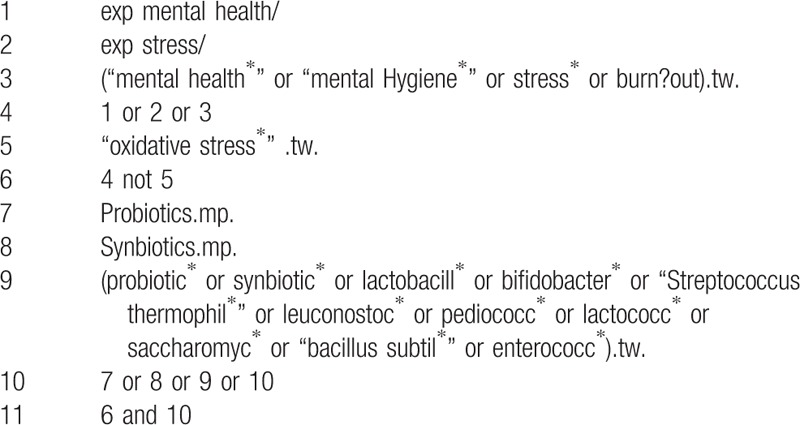
PsycINFO search strategy through Ovid.

**Table 5:**
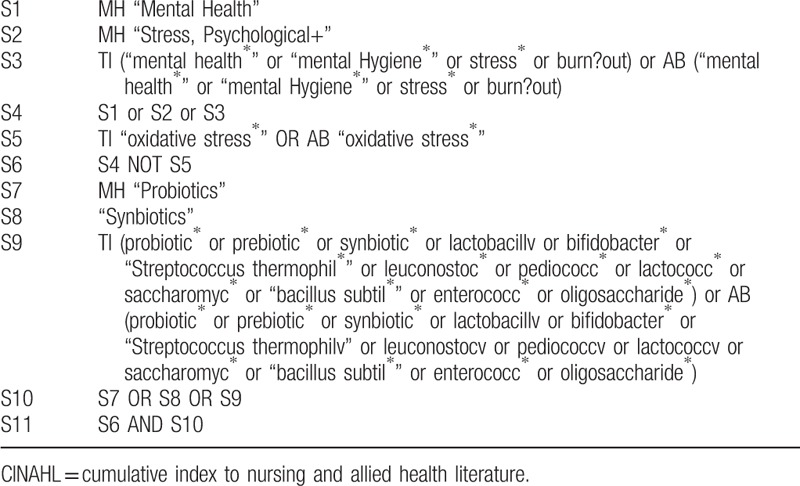
CINAHL search strategy through EBSCOhost.

### 2.8 Data collection and analysis

#### 2.8.1 Study selection

**Figure 1:**
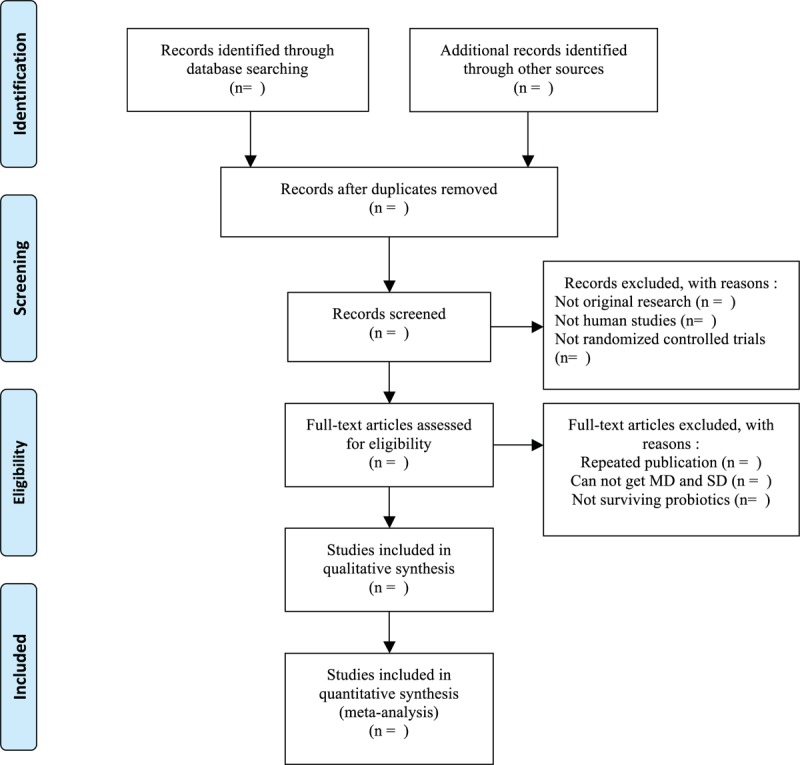
Flow diagram of the study selection process. Explains the specific process of the selection procedure with a PRISMA flow chart. PRISMA = preferred reporting items for systematic reviews and meta-analyses.

The retrieved literature will be imported into EndnoteX7 and the duplicate data will be removed. Preliminary screening of the literature will be conducted by 2 authors independently through reading the title and abstract. The full text of these potentially eligible studies will be retrieved and independently assessed for eligibility by those 2 authors. Any disagreement between them over the eligibility of particular studies will be resolved through discussion with a third reviewer. The specific process of the selection procedure is presented in a preferred reporting items for systematic reviews and meta-analyses flow chart (Fig. 1).

#### 2.8.2 Data extraction

A standardized, pre-piloted form will be used to extract data from the included studies for assessment of applicability of the data extraction form. Extracted information will include: study design, study population and participant demographics and baseline characteristics, details of the intervention and control conditions, study methodology, recruitment and study completion rates, measurement methods, measurement times and results for each outcome measure included, as well as information for assessment of the risk of bias. Two review authors will extract data independently, and discrepancies will be identified and resolved through discussion (with a third author when necessary).

#### 2.8.3 Risk of bias assessment

Review Manager V5.3.5 software will be used for all bias risk assessments. The risk of bias in the included study will be assessed by considering the following characteristics:

(1) Random sequence generation (selection bias);
(2) Allocation concealment (selection bias);
(3) Blinding of participants and personnel (performance bias);
(4) Blinding of outcome assessment (detection bias);
(5) Incomplete outcome data (attrition bias);
(6) Selective reporting (reporting bias);
(7) Other bias.

Disagreements between the review authors over the risk of bias in particular studies will be resolved by discussion, with involvement of a third review author when necessary.

#### 2.8.4 Measures of treatment effect

For continuous variable, if the measurement tool is same, we will use the mean difference with 95% confidence intervals (CIs) to analyze the treatment effect. If the measurement tool is different, we will use standardized mean difference to eliminate the effects of different measurement units of the multiple studies. For dichotomous variable, we will use the relative risk with 95% CIs to analyze the effects of treatment.

#### 2.8.5 Dealing with missing data

If the research report cannot provide all data in the data extraction form, we will try to contact the corresponding author or first author to obtain the data by e-mail. If the data cannot be obtained, the available data will be analyzed.

#### 2.8.6 Assessment of heterogeneity

We will use tests for heterogeneity to assess the heterogeneity of the statistics of multiple studies. If *I*^2^ value is less than 50% and *P* value is >.10, it is considered that the heterogeneity between multiple studies is acceptable. A range of 0% to 40% of *I*^2^ indicates the heterogeneity might not be important; a range of 30% to 60% indicates possible moderate heterogeneity among multiple studies; a range of 50% to 90% indicates substantial heterogeneity; a range of 75% to 100% indicates considerable heterogeneity. We will select models based on the heterogeneity between studies. If heterogeneity exists between multiple studies, we will perform sensitivity analysis and subgroup analysis to process heterogeneity and analyze heterogeneity sources.

#### 2.8.7 Subgroup analysis

It should be noted that this is a qualitative synthesis and while subgroup analyses may be undertaken. It is not possible to specify the groups in advance. If the necessary data are available, subgroup analyses might be done according to the age of participants, length of intervention, or the type of probiotics (including single-strain probiotics formulation and multi-strain probiotics formulation).

#### 2.8.8 Sensitivity analysis

We will eliminate the studies with more than 1 risk of high bias, or 2 or more risks of unknown bias by item. It will be observed that whether the quality of the study will affect the results of the study.

#### 2.8.9 Assessment of reporting biases

If more than 10 studies are included, we will use Review Manager V5.3.5 software to assess potential reporting bias. If there is a clear reporting bias, we will assess the impact of bias on the outcome.

## 3 Discussion

As we are concerned, excessive stress is threatening our physical and mental health and triggering more and more public health problems.^[26,27]^ As an important type of “spiritual microbes,” probiotics show good prospects in relieving stress and preventing stress-related health problems. Although many animal experiments and clinical studies have been conducted, the efficacy of probiotic supplements for relieving stress has not been scientifically and systematically evaluated. The purpose of this systematic review is to assess the effectiveness and safety of probiotics in relieving stress in healthy populations, and we hope that this study will provide additional evidence. Through preliminary search, we found that there are still many clinical studies underway. These studies are very important for the conclusion, so we consider updating the systematic review after 5 years.

## Author contributions

**Conceptualization:** Ning Zhang, Weiguang Wang, Shuangqing Zhai.

**Data curation:** Ning Zhang, Yanan Zhang, Menglin Li.

**Formal analysis:** Xing Liao, Yanan Zhang, Menglin Li.

**Investigation:** Yanan Zhang, Menglin Li.

**Methodology:** Xing Liao, Weiguang Wang.

**Project administration:** Shuangqing Zhai, Xing Liao.

**Software:** Ning Zhang.

**Supervision:** Shuangqing Zhai, Xing Liao.

**Validation:** Shuangqing Zhai, Xing Liao.

**Visualization:** Shuangqing Zhai, Ning Zhang.

**Writing – original draft:** Ning Zhang, Shuangqing Zhai, Yanan Zhang.

**Writing – review and editing:** Shuangqing Zhai, Xing Liao, Ning Zhang.
